# Genetic Incorporation of Unnatural Amino Acids into Proteins in *Mycobacterium tuberculosis*

**DOI:** 10.1371/journal.pone.0009354

**Published:** 2010-02-22

**Authors:** Feng Wang, Scott Robbins, Jiantao Guo, Weijun Shen, Peter G. Schultz

**Affiliations:** 1 Department of Chemistry and the Skaggs Institute for Chemical Biology, The Scripps Research Institute, La Jolla, California, United States of America; 2 Genomics Institute of the Novartis Research Foundation, San Diego, California, United States of America; University of California Merced, United States of America

## Abstract

New tools are needed to study the intracellular pathogen *Mycobacterium tuberculosis* (*Mtb*), the causative agent of tuberculosis (TB), to facilitate new drug discovery and vaccine development. We have developed methodology to genetically incorporate unnatural amino acids into proteins in *Mycobacterium smegmatis*, BCG and *Mtb*, grown both extracellularly in culture and inside host cells. Orthogonal mutant tRNA^Tyr^/tyrosyl-tRNA synthetase pairs derived from *Methanococcus jannaschii* and evolved in *Escherichia coli* incorporate a variety of unnatural amino acids (including photocrosslinking, chemically reactive, heavy atom containing, and immunogenic amino acids) into proteins in response to the amber nonsense codon. By taking advantage of the fidelity and suppression efficiency of the *Mj*tRNA/pIpaRS pair in mycobacteria, we are also able to use *p*-iodophenylalanine to induce the expression of proteins in mycobacteria both extracellularly in culture and inside of mammalian host cells. This provides a new approach to regulate the expression of reporter genes or mycobacteria endogenous genes of interest. The establishment of the unnatural amino acid expression system in *Mtb*, an intracellular pathogen, should facilitate studies of TB biology and vaccine development.

## Introduction

Tuberculosis (TB) remains among the most deadly diseases, causing more than two million deaths each year [1], [2]. Despite a worldwide effort for more effective *Mtb* chemotherapies and a more efficacious TB vaccine remain illusive [3]. New tools are needed to study the intracellular pathogen *Mycobacterium tuberculosis* (*Mtb*), the causative agent of tuberculosis (TB), to facilitate new drug discovery and vaccine development. Previously, we developed a method that makes it possible to genetically encode unnatural amino acids with diverse physical, chemical, and biological properties in *Escherichia coli* (*E. coli*), yeast, and mammalian cells [4], [5], [6], [7], [8], [9], [10], [11], [12], [13]. This method is based on the generation of an orthogonal tRNA/aminoacyl-tRNA synthetase (aaRS) pair that allows the site-selective incorporation of unnatural amino acids into proteins in response to unique nonsense and frameshift codons. The genetic incorporation of amino acids containing fluorophores, photocaged groups, photocrosslinkers, metal ion chelating groups and others at specific sites of proteins has provided a useful tool to explore protein structure and function *in vitro* and *in vivo*. Here we expand this approach to mycobacteria, and genetically incorporate five unnatural amino acids (including the photocrosslinkers *p*-azidophenylalanine [pAzpa] and *p*-benzoylphenylalanine [pBpa], the immunogenic amino acid *p*-nitrophenylalanine [pNO_2_pa], the heavy atom containing amino acid *p*-iodophenylalanine [pIpa], and the chemically reactive amino acid *p*-boronophenylalanine [pBO_2_pa]. Moreover, we demonstrate the applicability of this approach to the study of *Mtb* grown both extracellularly in culture and inside of mammalian host cells.

## Results and Discussion

To genetically encode unnatural amino acids in mycobacteria we adapted the existing mutant tRNA/aminoacyl-tRNA synthetase (aaRS) pairs from *Methanococcus jannaschii* (*M. jannaschii*) that were evolved in *Escherichia coli* (*E. coli*) to incorporate a wide array of unnatural amino acids with good efficiency and high fidelity. It has been found that the major recognition elements of *M. jannaschii* tRNA^Tyr^ (*Mj*tRNA), including the discriminator base A73 and the first base pair C1-G72, differ from those of *E. coli* so that the *M. jannaschii* tRNA-aaRS pairs do not cross-react with the endogenous *E. coli* tRNAs or aminoacyl-tRNA synthetases[4], [14], [15]. Mycobacteria and *E. coli* are both eubacteria and are phylogenetically closer to each other than to archaea; they also use similar transcriptional and translational systems. Indeed the predicted structures of the tRNA^Tyr^s of *Mtb* and *M. smegmatis* revealed identity elements similar to those of *E. coli*, including the discriminator base A, the first base pair G-C, and variable arm (although it is shorter than that in *E. coli* tRNA), which are distinct from those of *Mj*tRNA. Therefore, we felt it likely that the mutant tRNA-aaRS pairs of *M. jannaschii* would be orthogonal in mycobacteria as well.

**Figure 1:**
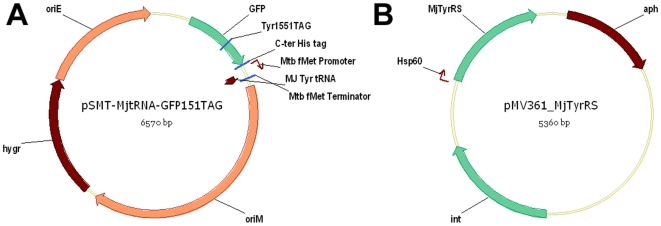
Plasmid maps. **A. pSMT-*Mj*tRNA-GFP151TAG; B. pMV361-*Mj*TyrRS.**

To transcribe the exogenous *Mj*tRNA in mycobacteria, we used the *Mycobacterium tuberculosis* (*Mtb*) initiator tRNA (*metU*) promoter (from −40 to +1) and terminator which have been found to strongly drive gene expression in both fast and slow growing mycobacteria [16]. The tRNA gene and regulatory elements were inserted into a *Mtb*-*E. coli* shuttle vector to create plasmid pSMT-*Mj*tRNA. We then inserted a GFP(Tyr151TAG) mutant into the same plasmid under the *Mtb hsp60* promoter to afford plasmid pSMT-*Mj*tRNA-GFP151TAG (Figure 1A). This GFP151TAG mutant provides a rapid qualitative assay of amber-suppression efficiency in *E. coli*; it has been demonstrated previously that at position Tyr151 introduction of unnatural amino acids does not interfere with the fluorophore or affect the correct folding of the protein [17]. To facilitate protein purification, a six-histidine tag was fused at the C- terminus. Next we inserted the *M. jannaschii* tyrosyl-tRNA synthetase (*Mj*TyrRS) into an integrating *Mtb*-*E. coli* shuttle vector pMV361 under control of the *Mtb hsp60* promoter (Figure 1B). We then assayed the suppression of the nonsense amber codon in GFP(Tyr151TAG) mutant in *Mycobacterium smegmatis* (*M. smegmatis*) by monitoring green fluorescence. Due to the low transformation efficiency of mycobacteria, the two plasmids were transformed into *M. smegmatis* sequentially and selected on hygromycin and kanamycin. As control, pSMT-*Mj*tRNA-GFP151TAG was transformed into *M. smegmatis* and selected on hygromycin. Single colonies of both single and double transformants were picked and cultured for two days. Green fluorescence was only observed in colonies containing two plasmids, indicating that the nonsense amber codon encoded in GFP is only suppressed in the presence of the *Mj*tRNA-TyrRS pair. These results confirmed that *M. jannaschii* tRNA-aaRS pair is functional and *Mj*tRNA is orthogonal inside mycobacteria.

**Figure 2:**
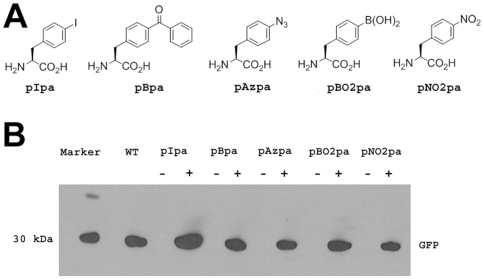
Amber suppression of five *Mj*tRNA^Tyr^-*Mj*TyrRS pairs in *M. smegmatis*. A. Chemical structures of the five unnatural amino acids; B. Western blot analysis of GFP expression in *M. smegmatis* cells that were cotransformed with pSMT-*Mj*tRNA-GFP151TAG and the pMV361-*Mj*TyrRS variants. The first lane is Novex Sharp prestained marker. The second lane is empty. The third lane is expression of GFP151TAG suppressed by wild type *Mj*TyrRS. The following lanes are expression of GFP151TAG suppressed by *Mj*TyrRS variants in the presence (+) or absence (−) of unnatural amino acids. A 40 µg aliquot of cell lysate for each reaction was analyzed with anti-His-HRP.

We next examined the ability to genetically incorporate five unnatural amino acids (pAzpa, pBpa, pNO_2_pa, pIpa, and pBO_2_pa) into the amber nonsense mutant GFP in mycobacteria (Figure 2A). Each of the five *Mj*TyrRS variants was inserted into the pMV361 vector and co-transformed with pSMT-*Mj*tRNA-GFP151TAG into *M. smegmatis*. We then tested the expression of the full length GFP in the presence and absence of the corresponding unnatural amino acid. After the cell density reached OD_600_ 0.4, 2 mM of either pAzpa, pBpa, pNO_2_pa, pIpa, or pBO_2_pa was added to the culture. After 16 hours, a fluorescence signal was detected in all five cultures in the presence of the corresponding unnatural amino acids, indicating the expression of the full length GFP; little or no fluorescence was observed in the absence of the unnatural amino acids. As controls, we also cultured *M. smegmatis* transformed with either pSMT-*Mj*tRNA-GFP151TAG plasmid or the pMV361-*Mj*TyrRS varient/pSMT-GFP151TAG (which does not encode *Mj*tRNA, but only the GFP Tyr151TAG mutant) pair and tested the expression of GFP in the presence and absence of unnatural amino acids. No fluorescence signal was detected under either condition, indicating that the *Mj*TyrRS mutant and *Mj*tRNA are required to incorporate the unnatural amino acid. Western blot analysis of full length GFP also confirmed the efficient suppression of the TAG codon in the GFP reporter in the presence of the mutant *Mj*TyrRS-*Mj*tRNA pair (Figure 2B). Although all five unnatural amino acids were incorporated into GFP, the expression levels of GFP varied for the different unnatural amino acids, mostly likely due to differences in the efficiency of amino acid charging and/or amino acid uptake. pIpa afforded the highest yield of GFP (0.6 mg/L, 9% of wild type GFP yield). We also characterized the GFP mutants expressed in this system by mass spectrometry. The His-tagged GFP mutants were expressed in *M. smegmatis* in the presence of the unnatural amino acids for 16 hours and purified by Ni-NTA column. LC-MS analysis of the purified GFP mutants confirmed the selective incorporation of the unnatural amino acid in all cases (Figure S1, Figure S2, Figure S3, Figure S4 and Figure S5).

**Figure 3:**
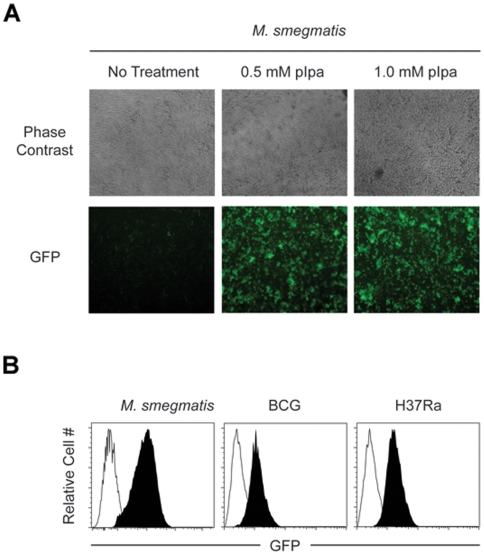
Regulation of GFP expression in *M. smegmatis*, BCG, and *Mtb* H37Ra by unnatural amino acid mediated nonsense codon suppression. A. pIpa-GFP151TAG-*M. smegmatis* in 96 well flat-bottom plates was incubated with the indicated concentration of pIpa for 16 hrs. Representative phase contrast and GFP fluorescence images for each condition are shown; B. pIpa-GFP151TAG-*M. smegmatis*, pIpa-GFP151TAG-BCG, and pIpa-GFP151TAG-H37Ra were incubated in the absence (open histograms) or presence (filled historgrams) of 1 mM pIpa for 16 hrs. The GFP fluorescent intensity of the bacteria populations was determined by flow cytometry.

To determine if this unnatural amino acid expression system can be used in slow growing mycobacteria, we transformed the pMV361-*Mj*pIpaRS/pSMT-*Mj*tRNA-GFP151TAG pair into BCG and *Mtb* strain H37Ra. Both BCG and *Mtb* H37Ra strains carrying the two plasmids grow at normal rates on 7H11 agar plates or in 7H9 liquid medium. Fluorescence analysis using flow cytometry indicated no expression of GFP in the absence of pIpa. Expression of amber nonsense mutant GFP was then induced by addition of 0.5 mM and 1 mM pIpa to liquid cultures of BCG and *Mtb* at OD 0.4, respectively. After 16 hours, strong fluorescent signals were detected and GFP expression was confirmed by western blot (Figure S6). This is consistent with what we observed in the *M. smegmatis* expression system, and demonstrates the applicability of this technology to slow growing mycobacterial species. Importantly, the use of flow cytometry for the analysis of GFP expression allowed us to demonstrate that GFP was expressed in each individual bacterial cell within the cultures and not just within a subpopulation of cells (Figure 3).

**Figure 4:**
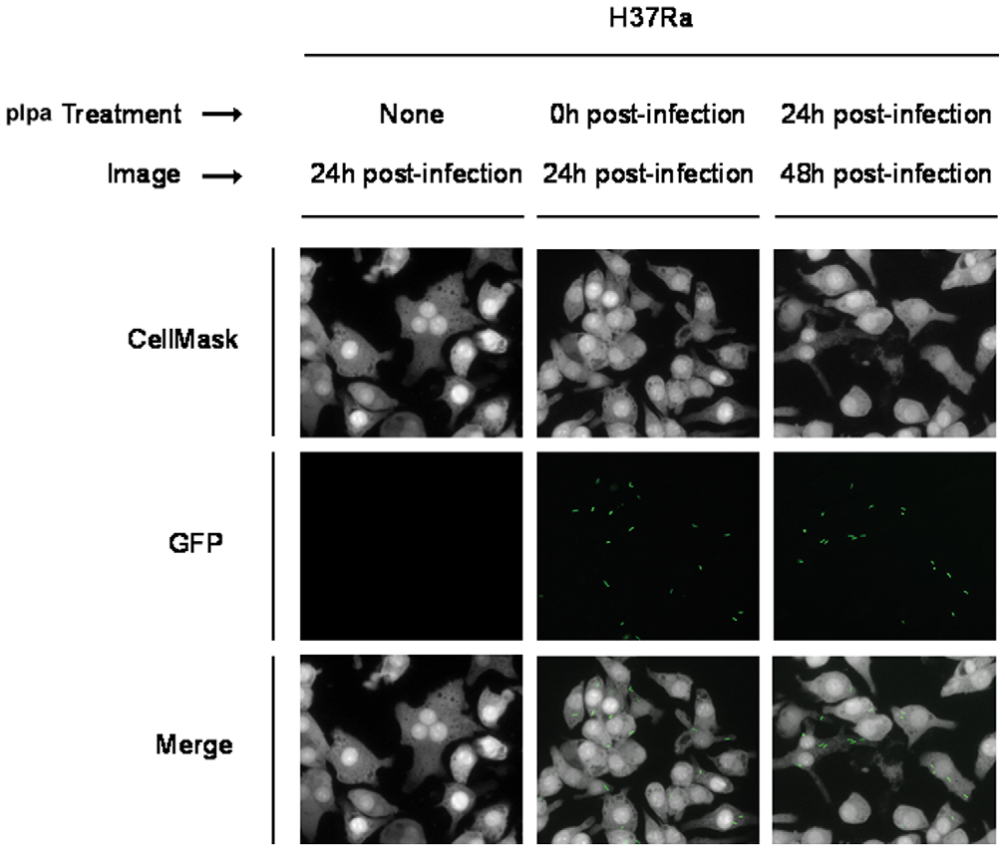
Regulation of GFP expression by pIpa incorporation in intra-macrophage *Mtb* H37Ra. J774 cells were infected with pIpa-GFP151TAG-H37Ra, and cultured in the absence or presence of 1mM pIpa for the indicated time-periods post-infection. Infected J774 cells were fixed and stained with CellMask at the indicated time-points post-infection. J774 cells and intracellular H37Ra were visualized by fluorescent microscopy. Representative images for each condition are shown.

We next determined if protein expression could be regulated by a similar strategy in mycobacteria growing inside macrophages. To test the ability of the pIpa-induction system to function under intracellular conditions we infected J774 cells with pIpa-GFP151TAG-H37Ra *in vitro* and cultured the infected macrophages in the absence or presence of 1 mM pIpa for 24 hours. As shown in Figure 4, we could detect GFP^+^ pIpa-GFP151TAG-H37Ra within J774, but only in the presence of pIpa. Within the same experiment we also delayed the addition of pIpa until 24 hours post-macrophage infection and assayed for the expression of GFP in pIpa-GFP151TAG-H37Ra after an additional 24 hours. As with the immediate addition of pIpa, we could detect GFP^+^ bacteria within the macrophages only in the presence of pIpa (Figure 4). The results of a CFU assay performed in parallel confirmed the viability of H37Ra bacteria inside J774 cells. We then infected J774 cells with *M. smegmatis* carrying the same *Mj*pIpaRS/pSMT-*Mj*tRNA-GFP151TAG pair under the same conditions. 1 mM pIpa was added to induce the expression of GFP 24 hours post infection. We could not detect any green fluorescence 24 hour after induction, suggesting that all *M. smegmatis* were killed intracellularly before the induction of GFP expression. Indeed, no viable *M. smegmatis* cells were found by CFU assay. These results demonstrate that the viability of intracellular mycobacteria can be correlated with the green fluorescent signal by using the pIpa induced reporter system. pIpa can tightly control the expression of the amber mutant GFP in mycobacteria growing inside the host cells, which makes this pIpa-induced reporter system a useful tool (in addition to shorter-lived fluorescent protein variants) to assay the viability of intracellular mycobacteria. Furthermore, it is possible to expand this technology to the regulation of other endogenous genes of interest in mycobacterial species after their infection of macrophages.

It is worth noting that of the total 4043 ORFs in the *Mycobacterium tuberculosis* (*Mtb*) H37Ra genome, there are 1194 (29.5%) ORFs that only use TAG as the stop codon to terminate protein translation. This contrasts with the 365 genes in *E. coli* that use only the amber stop codon. These amber stop codons may be suppressed in the presence of the amber suppressor *Mj*tRNA/aminoacyl-tRNA synthetase pair and the corresponding unnatural amino acid. However, the suppression efficiency of this pair in mycobacteria is low (the yields of mutant protein containing an unnatural amino acid are less than 9% that of wild type protein) indicating that the suppressor tRNA does not efficiently compete with release factor in this system. Thus, for those genes ending with the amber stop codon, most of translated protein will be terminated normally and partial suppression of the amber stop codon by the *Mj*tRNA/aminoacyl-tRNA synthetase pair will likely not affect the normal growth of *Mtb*. Indeed, as we demonstrate in supplementary Figure 7 – the growth rate of *Mtb* transformed with *Mj*tRNA/pIpaRS is indistinguishable from WT *Mtb* in the presence or absence of 1mM pIpa (Figure S7). However, we have not carried out a systematic study to determine what fraction of protein terminated with an amber codon generates any significant amount of longer readthrough product containing the unnatural amino acid. This would not affect biophysical or biochemical studies of purified proteins. Nor would it likely affect photocrosslinking studies in intact cells as the mutant protein and any crosslinked interacting partners would probably be isolated by affinity methods. However, suppression of normal termination codons could affect studies of mutant proteins containing for example fluorescent amino acids in living cells (in this case leading to a high background). Readthrough of endogenous stop codons may also be useful by providing a convenient mechanism for tagging proteins in the cell with C-terminal affinity labels. Future work will focus on this aspect of the methodology in both prokaryotic and eukaryotic cells.

In summary, by utilizing suitable mycobacterial promoters, *Mj*tRNA and *Mj*TyrRS variants were functionally expressed in *M. smegmatis*, BCG and *Mtb*. Five unnatural amino acids with useful physical, chemical, and/or biological properties have been incorporated into proteins in mycobacteria using these orthogonal pairs. Among them, the photocrosslinkers pBpa and pAzpa enable one to probe protein-protein or protein-nucleic acid interactions inside *Mtb* or between *Mtb* and host cells. pBO_2_pa can form reversible covalent complexes with diols, amino alcohols, and amino acids [18], and may be useful for surface protein modification in live mycobacteria. pNO_2_pa, an immunogenic amino acid, has the potential to boost BCG immunogenicity and create novel TB vaccines [19]. By taking advantage of the fidelity and suppression efficiency of the *Mj*tRNA/pIpaRS pair in mycobacteria, we are also able to use pIpa to induce the expression of proteins in mycobacteria both extracellularly in culture and inside of mammalian host cells. This provides an approach to regulate the expression of reporter genes or mycobacteria endogenous genes of interest. The establishment of the unnatural amino acid expression system in *Mtb*, an intracellular pathogen, should facilitate the studies of TB biology and vaccine development.

## Materials and Methods

### Bacteria Strains and Growth Conditions

All mycobacterial strains were grown on Middlebrook 7H11 agar at 37°C, 5% CO_2_-95% air atmosphere. For broth culture, *Mtb* H37Ra and BCG were grown in 7H9 medium supplemented with glycerol (0.5%, vol/vol) and OADC supplement. *M. smegmatis* was grown in 7H9 medium supplemented with glycerol (0.5%, vol/vol) and ADS supplement. All liquid cultures were supplemented with 0.05% Tween 80.

### Construction of Plasmids

The pSMT-*Mj*tRNA was constructed by PCR amplification of the *Mj*tRNA gene with primers flanked by the *Mtb* fMet promoter and terminator sequences. The PCR product was digested with NdeI and HindIII and inserted into a vector derived from plasmid pSMT3. The GFP151TAG gene from plasmid pET22b-GFP151TAG was inserted into the BamHI and NdeI sites of pSMT-*Mj*tRNA to generate pSMT-*Mj*tRNA-GFP151TAG. Similarly, GFP151TAG was inserted into the BamHI and HindIII sites to afford pSMT-GFP151TAG (Extended Methods S1). The coding sequences of *Mj*TyrRS variants were ligated into the BamHI and HindIII sites of pMV361 to generate pMV361-pIpaRS, pMV361-pAzpaRS, pMV361-pBpaRS, pMV361-pNO_2_paRS, and pMV361-pBO_2_paRS.

### FACS Analysis

LSRII and FACS Diva software (both Beckton Dickenson) were used to acquire data to determine the expression of GFP in pIpa-GFP151TAG-*M. smegmatis*, pIpa-GFP151TAG-BCG, and pIpa-GFP151TAG-H37Ra by flow cytometry. Data was analyzed using FlowJo software (TreeStar).

### Microscopy

At the time-points indicated in the text, infected J774 cells were fixed and stained with CellMask Blue (invitrogen) per manufacturer's instructions. J774 cells and intracellular H37Ra were visualized on a Nikon fluorescent microscope. Images were captured using MetaMorph software and figure images generated in Photoshop (Adobe).

### Macrophage Infections

pIpa-GFP151TAG-H37Ra was grown to an OD_600_ of 0.4–0.6. In order to obtain a single cell suspension for use in the infection assay, the following procedure was performed. Bacteria were centrifuged and washed twice in PBS, re-suspended in media (no additives), and sonicated for 10 sec in a cuphorn sonicator, twice. Sonicated bacteria were passed through a 26 gauge needle and then a 5 micron filter. The OD_600_ for the single cell bacterial suspension was then determined and the number of bacteria present quantified. 2X media containing FCS was then added to the single cell suspension of bacteria to bring them to the appropriate concentration for the infection assay. J774 cells (1×10^5^/well in a 96 well plate) were then infected with pIpa-GFP151TAG-H37Ra at an MOI of 5. Infection was allowed to proceed for 4 hours, after which the media was aspirated and wells were washed 3 times with fresh media. After the final wash, J774 growth media containing 10 µg/ml amikacin (to kill any remaining extracellular bacteria) was added to each well.

## Supporting Information

Extended Methods S1 Extended Methods
(0.03 MB DOC)

Figure S1 A. Deconvoluted ESI-MS spectra of the GFP mutant Tyr151→pIpa. Expected mass is 27815 Da; observed masses are 27816 Da and 27947 Da (with N-terminal methionine). B. Full ESI-MS spectra of the GFP mutant Tyr151→pIpa.
(0.05 MB PDF)

Figure S2 Deconvoluted ESI-MS spectra of the GFP mutant Tyr151→pAzpa. Expected mass is 27733 Da; observed masses are 27730 Da and 27861 Da (with N-terminal methionine); the observed mass 27706 corresponds to the mass of GFP mutant Tyr151→pAzpa after the azido group is reduced to amine by photo activation. The mass peak 27810 is not corresponding to the natural amino acid incorporated GFP and is most likely impurity. B. Full ESI-MS spectra of the GFP mutant Tyr151→pAzpa.
(0.06 MB PDF)

Figure S3 Deconvoluted ESI-MS spectra of the GFP mutant Tyr151→pBO2pa. Expected mass is 27718 Da; observed masses are 27720 Da and 27850 Da (with N-terminal methionine). B. Full ESI-MS spectra of the GFP mutant Tyr151→pBO2pa.
(0.06 MB PDF)

Figure S4 Deconvoluted ESI-MS spectra of the GFP mutant Tyr151→pNO2pa. Expected mass is 27737 Da; observed masses are 27738 Da and 27867 Da (with N-terminal methionine). The mass peak 27810 is not corresponding to the natural amino acid incorporated GFP and is most likely impurity. B. Full ESI-MS spectra of the GFP mutant Tyr151→pNO2pa.
(0.06 MB PDF)

Figure S5 Deconvoluted ESI-MS spectra of the GFP mutant Tyr151→pBpa. Expected mass is 27796 Da; observed masses are 27795 Da and 27927 Da (with N-terminal methionine). B. Full ESI-MS spectra of the GFP mutant Tyr151→pBpa.
(0.06 MB PDF)

Figure S6 Western blot analysis of GFP expression in Mtb and BCG cells that were cotransformed with pSMT-MjtRNA-GFP115TAG and pMV361-MjpIpaRS. The first lane is wild type GFP expressed in M. smegmatis. The following lanes are expression of GFP151TAG suppressed by MjpIpaRS in the presence (+) and absence (−) of 1 mM pIpa. A 60 µg aliquot of cell lysate for each reaction (10 µg for the WT) was analyzed with anti-His-HRP.
(0.08 MB PDF)

Figure S7 Growth of pIpa-GFP151TAG-H37Ra. A. Growth rates of wild type H37Ra (black dots) and strain pIpa-GFP151TAG-H37Ra (red dots) in 7H9 media. B. Growth rates of wild type H37Ra (black dots) and strain pIpa-GFP151TAG-H37Ra (red dots) in 7H9 media treated with 1 mM pIpa.
(0.03 MB PDF)

## References

[1] Dye C (2006). Global epidemiology of tuberculosis.. Lancet.

[2] Harries AD, Dye C (2006). Tuberculosis.. Ann Trop Med Parasitol.

[3] Fine PE (1995). Variation in protection by BCG: implications of and for heterologous immunity.. Lancet.

[4] Wang L, Brock A, Herberich B, Schultz PG (2001). Expanding the genetic code of Escherichia coli.. Science.

[5] Chin JW, Cropp TA, Anderson JC, Mukherji M, Zhang Z (2003). An expanded eukaryotic genetic code.. Science.

[6] Chin JW, Martin AB, King DS, Wang L, Schultz PG (2002). Addition of a photocrosslinking amino acid to the genetic code of Escherichiacoli.. Proc Natl Acad Sci U S A.

[7] Deiters A, Cropp TA, Mukherji M, Chin JW, Anderson JC (2003). Adding amino acids with novel reactivity to the genetic code of Saccharomyces cerevisiae.. J Am Chem Soc.

[8] Lee HS, Guo J, Lemke EA, Dimla RD, Schultz PG (2009). Genetic Incorporation of a Small, Environmentally Sensitive, Fluorescent Probe into Proteins in Saccharomyces cerevisiae.. J Am Chem Soc.

[9] Lee HS, Spraggon G, Schultz PG, Wang F (2009). Genetic incorporation of a metal-ion chelating amino acid into proteins as a biophysical probe.. J Am Chem Soc.

[10] Liu CC, Schultz PG (2006). Recombinant expression of selectively sulfated proteins in Escherichia coli.. Nat Biotechnol.

[11] Liu W, Brock A, Chen S, Schultz PG (2007). Genetic incorporation of unnatural amino acids into proteins in mammalian cells.. Nat Methods.

[12] Wang J, Xie J, Schultz PG (2006). A genetically encoded fluorescent amino acid.. J Am Chem Soc.

[13] Xie J, Liu W, Schultz PG (2007). A genetically encoded bidentate, metal-binding amino acid.. Angew Chem Int Ed Engl.

[14] Wang L, Magliery TJ, Liu DR, Schultz PG (2000). A New Functional Suppressor tRNA/Aminoacyl-tRNA Synthetase Pair for the in Vivo Incorporation of Unnatural Amino Acids into Proteins.. J Am Chem Soc.

[15] Wang L, Schultz PG (2001). A general approach for the generation of orthogonal tRNAs.. Chem Biol.

[16] Vasanthakrishna M, Kumar NV, Varshney U (1997). Characterization of the initiator tRNA gene locus and identification of a strong promoter from Mycobacterium tuberculosis.. Microbiology.

[17] Ormo M, Cubitt AB, Kallio K, Gross LA, Tsien RY (1996). Crystal structure of the Aequorea victoria green fluorescent protein.. Science.

[18] Brustad E, Bushey ML, Lee JW, Groff D, Liu W (2008). A genetically encoded boronate-containing amino acid.. Angew Chem Int Ed Engl.

[19] Grunewald J, Tsao ML, Perera R, Dong L, Niessen F (2008). Immunochemical termination of self-tolerance.. Proc Natl Acad Sci U S A.

