# Supplementary material for: Genetic Incorporation of Unnatural Amino Acids into Proteins in Mycobacterium tuberculosis
Source: PLoS One. 2010 Feb 22;5(2):e9354. doi: 10.1371/journal.pone.0009354 (PMC2825273; doi:10.1371/journal.pone.0009354)
Supplement: Extended Methods S1 — Extended Methods (0.03 MB DOC) [file pone.0009354.s001.doc]

**Extended Methods**

**Sequence of the plasmid pSMT-MjtRNA-GFP151TAG**

ctagaggtgaccacaacgacgcgcccgctttgatcggggacgtctgcggccgaccatttacgggtcttgttgtcgttggcggtcatgggccgaacatactcacccggatcggagggccgaggacaaggtcgaacgaggggcatgacccggtgcggggcttcttgcactcggcataggcgagtgctaagaataacgttggcactcgcgaccggtgagtcgtaggtcgggacggtgaggccaggcccgtcgtcgcagcgagtggcagcgaggacaacttgagccgtccgtcgcgggcactgcgcccggccagcgtaagtagcggggttgccgtcacccggtgacccccggtttcatccccgatccggaggaatcacttccatatggtgagcaagggcgaggagctgttcaccggggtggtgcccatcctggtcgagctggacggcgacgtaaacggccacaagttcagcgtgtccggcgagggcgagggcgatgccacctatggcaagctgaccctgaagttcatctgcaccaccggcaagctgcccgtgccctggcccaccctcgtgaccaccctgacctacggcgtgcagtgcttcagccgctaccccgaccacatgaagcagcacgacttcttcaagtccgccatgcccgaaggctacgtccaggagcgcaccatcttcttcaaggacgacggcaactacaagacccgcgccgaggtgaagttcgagggcgacaccctggtgaaccgcatcgagctgaagggcatcgacttcaaggaggacggcaacatcctggggcacaagctggagtacaactacaacagccacaacgtctagatcatggccgacaagcagaagaacggcatcaaggtgaacttcaagatccgccacaacatcgaggacggcagcgtgcagctcgccgaccactaccagcagaacacccccatcggcgacggccccgtgctgctgcccgacaaccactacctgagcacccagtccgccctgagcaaagaccccaacgagaagcgcgatcacatggtcctgctggagttcgtgaccgccgccgggatcactctcggcatggacgagctgtacaaggctagccaccaccaccaccaccactgaacgcgtgatttggcgagcttcgtgcgtgttcggtagcctggcatttaccgaccggcggtagttcagcagggcagaacggcggactctaaatccgcatggcgctggttcaaatccggcccgccggaccagcggaaatgaccccagaggtgcgacctctggggtaagcttatcgataccgtcgacctcgagggggggcccggtacgtacccggggatcatcgagccgagaacgttatcgaagttggtcatgtgtaatcccctcgtttgaactttggattaagcgtagatacacccttggacaagccagttggattcggagacaagcaaattcagccttaaaaagggcgaggcctgcggtggtggaacaccgcagggcctctaaccgctcgacgcgctgcaccaaccagcccgcgaacggctggcagccagcgtaaggcgcggctcatcgggcggcgttcgccacgatgtcctgcacttcgagccaagcctcgaacacctgctggtgtgcacgactcacccggttgttgacaccgcgcgcggccgtgcgggctcggtggggcggctgtgtcgcccttgccagcgtgagtagcgcgtacctcacctcgcccaacaggtcgcacacagccgattcgtacgccataaagccaggtgagcccaccagctccgtaagttcgggcgctgtgtggctcgtacccgcgcattcaggcggcagggggtctaacgggtctaaggcggcgtgtacggccgccacagcggctctcagcggcccggaaacgtcctcgaaacgacgcatgtgttcctcctggttggtacaggtggttgggggtgctcggctgtcgctggtgttccaccaccagggctcgacgggagagcgggggagtgtgcagttgtggggtggcccctcagcgaaatatctgacttggagctcgtgtcggaccatacaccggtgattaatcgtggtctactaccaagcgtgagccacgtcgccgacgaatttgagcagctctggctgccgtactggccgctggcaagcgacgatctgctcgaggggatctaccgccaaagccgcgcgtcggccctaggccgccggtacatcgaggcgaacccaacagcgctggcaaacctgctggtcgtggacgtagaccatccagacgcagcgctccgagcgctcagcgcccgggggtcccatccgctgcccaacgcgatcgtgggcaatcgcgccaacggccacgcacacgcagtgtgggcactcaacgcccctgttccacgcaccgaatacgcgcggcgtaagccgctcgcatacatggcggcgtgcgccgaaggccttcggcgcgccgtcgacggcgaccgcagttactcaggcctcatgaccaaaaaccccggccacatcgcctgggaaacggaatggctccactcagatctctacacactcagccacatcgaggccgagctcggcgcgaacatgccaccgccgcgctggcgtcagcagaccacgtacaaagcggctccgacgccgctagggcggaattgcgcactgttcgattccgtcaggttgtgggcctatcgtcccgccctcatgcggatctacctgccgacccggaacgtggacggactcggccgcgcgatctatgccgagtgccacgcgcgaaacgccgaattcccgtgcaacgacgtgtgtcccggaccgctaccggacagcgaggtccgcgccatcgccaacagcatttggcgttggatcacaaccaagtcgcgcatttgggcggacgggatcgtggtctacgaggccacactcagtgcgcgccagtcggccatctcgcggaagggcgcagcagcgcgcacggcggcgagcacagttgcgcggcgcgcaaagtccgcgtcagccatggaggcattgctatgagcgacggctacagcgacggctacagcgacggctacaaccggcagccgactgtccgcaaaaagcggcgcgtgaccgccgccgaaggcgctcgaatcaccggactatccgaacgccacgtcgtccggctcgtggcgcaggaacgcagcgagtggctcgccgagcaggctgcacgccgcgaacgcatccgcgcctatcacgacgacgagggccactcttggccgcaaacggccaaacatttcgggctgcatctggacaccgttaagcgactcggctatcgggcgaggaaagagcgtgcggcagaacaggaagcggctcaaaaggcccacaacgaagccgacaatccaccgctgttctaacgcaattggggagcgggtgtcgcgggggttccgtggggggttccgttgcaacgggtcggacaggtaaaagtcctggtagacgctagttttctggtttgggccatgcctgtctcgttgcgtgtttcgttgcgcccgttttgaataccagccagacgagacggggttctacgaatcttggtcgataccaagccatttccgctgaatatcggggagctcaccgccagaatcggtggttgtggtgatgtacgtggcgaactccgttgtagtgcctgtggtggcatccgtggccactctcgttgcacggttcgttgtgccgttacaggccccgttgacagctcaccgaacgtagttaaaacatgctggtcaaactaggtttaccaacgatacgagtcagctcatctagggccagttctaggcgttgttcgttgcgcggttcgttgcgcatgtttcgtgtggttgctagatggctccgcaaccacacgcttcgaggttgagtgcttccagcacgggcgcgatccagaagaacttcgtcgtgcgactgtcctcgttgatccttgccgagctgggatggaagctcggccgaccaccctggaggagatgatcgaggatgccagggcctttcacgcccgccgctgctgagcgtccgccgccgggcccgcaccgccgtcggccggcccgctccgggctcgcagcagcgggcttcggcgcgggcccggggctcccgagcgcgggcggggctccgggcggccgccgggggccgggggcggcgccgggcggcccggggcgtcaggcgccgggggcggtgtccggcggcccccagaggaactgcgccagttcctccggatcggtgaagccggagagatccagcggggtctcctcgaacacctcgaagtcgtgcaggaaggtgaaggcgagcagttcgcgggcgaagtcctcggtccgcttccactgcgccccgtcgagcagcgcggccaggatctcgcggtcgccccggaaggcgttgagatgcagttgcaccaggctgtagcgggagtctcccgcatagacgtcggtgaagtcgacgatcccggtgacctcggtcgcggccaggtccacgaagatgttggtcccgtgcaggtcgccgtggacgaaccggggttcgcggccggccagcagcgtgtccacgtccggcagccagtcctccaggcggtccagcagccggggcgagaggtagccccacccgcggtggtcctcgacggtcgccgcgcggcgttcccgcagcagttccgggaagacctcggaatggggggtgagcacggtgttcccggtcagcggcaccctgtgcagccggccgagcacccggccgagttcgcgggccagggcgagcagcgcgttccggtcggtcgtgccgtccatcgcggaccgccaggtggtgccggtcatccggctcatcaccaggtagggccacggccaggctccggtgccgggccgcagctcgccgcggccgaggaggcggggcaccggcaccggggcgtccgccaggaccgcgtacgcctccgactccgacgcgaggctctccggaccgcaccagtgctcgccgaacagcttgatcaccgggtcgggctcgccgaccagtacggggttggtgctctcgccgggcacccgcagcaccggcggcaccggcagcccgagctcctccagggctcggcgggccagcggctcccagaattcctggtcgttccgcaggctcgcgtaggaatcatccgaatcaatacggtcgagaagtaacagggattcttgtgtcacagcggacctctattcacagggtacgggccggcttaattccgcacggccggtcgcgacacggcctgtccgcaccgcggatcaggcgttgacgatgacgggctggtcggccacgtcggggacgacggggagtcaggcaactatggatgaacgaaatagacagatcgctgagataggtgcctcactgattaagcattggtaactgtcagaccaagtttactcatatatactttagattgatttaaaacttcatttttaatttaaaaggatctaggtgaagatcctttttgataatctcatgaccaaaatcccttaacgtgagttttcgttccactgagcgtcagaccccgtagaaaagatcaaaggatcttcttgagatcctttttttctgcgcgtaatctgctgcttgcaaacaaaaaaaccaccgctaccagcggtggtttgtttgccggatcaagagctaccaactctttttccgaaggtaactggcttcagcagagcgcagataccaaatactgtccttctagtgtagccgtagttaggccaccacttcaagaactctgtagcaccgcctacatacctcgctctgctaatcctgttaccagtggctgctgccagtggcgataagtcgtgtcttaccgggttggactcaagacgatagttaccggataaggcgcagcggtcgggctgaacggggggttcgtgcacacagcccagcttggagcgaacgacctacaccgaactgagatacctacagcgtgagctatgagaaagcgccacgcttcccgaagggagaaaggcggacaggtatccggtaagcggcagggtcggaacaggagagcgcacgagggagcttccagggggaaacgcctggtatctttatagtcctgtcgggtttcgccacctctgacttgagcgtcgatttttgtgatgctcgtcaggggggcggagcctatggaaaaacgccagcaacgcggcctttttacggttcctggccttttgctggccttttgctcacatgttctttcctgcgttatcccctgattctgtggataaccgtattaccgcctttgagtgagctgataccgctcgccgcagccgaacgaccgagcgcagcgagtcagtgagcgaggaagcggaagagcgcccaatacgcaaaccgcctctccccgcgcgttggccgattcattaatgcagctggcacgacaggtttcccgactggaaagcgggcagtgagcgcaacgcaattaatgtgagttagctcactcattaggcaccccaggctttacactttatgcttccggctcgtatgttgtgtggaattgtgagcggataacaatttcacacaggaaacagctatgaccatgattaccagatctggctcgagctccaccgcggtggcggccgct

**Sequence of the plasmid pMV361-MjpIpaRS**

gctagccaacaaagcgacgttgtgtctcaaaatctctgatgttacattgcacaagataaaaatatatcatcatgaacaataaaactgtctgcttacataaacagtaatacaaggggtgttatgagccatattcaacgggaaacgtcttgctcgaggccgcgattaaattccaacatggatgctgatttatatgggtataaatgggctcgcgataatgtcgggcaatcaggtgcgacaatctatcgcttgtatgggaagccccatgcgccagagttgtttctgaaacatggcaaaggtagcgttgccaatgatgttacagatgagatggtcagactaaactggctgacggaatttatgcctcttccgaccatcaagcattttatccgtactcctgatgatgcatggttactcaccactgcgatccccgggaaaacagcattccaggtattagaagaatatcctgattcaggtgaaaatattgttgatgcgctggcagtgttcctgcgccggttgcattcgattcctgtttgtaattgtccttttaacagcgatcgcgtatttcgtctcgctcaggcgcaatcacgaatgaataacggtttggttgatgcgagtgattttgatgacgagcgtaatggctggcctgttgaacaagtctggaaagaaatgcataatcttttgccattctcaccggattcagtcgtcactcatggtgatttctcacttgataaccttatttttgacgaggggaaattaataggttgtattgatgttggacgagtcggaatcgcagaccgataccaggatcttgccatcctatggaactgcctcggtgagttttctccttcattacagaaacggctttttcaaaaatatggtattgataatcctgatatgaataaattgcagtttcatttgatgctcgatgagtttttctaatcagaattggttaattggttgtaacactggcagagcattacgctgacttgacgggacggcggctttgttgaataaatcgaacttttgctgagttgaaggatcagatcacgcatcttcccgacaacgcagaccgttccgtggcaaagcaaaagttcaaaatcaccaactggtccacctacaacaaagctctcatcaaccgtggctccctcactttctggctggatgatggggcgattcaggcctggtatgagtcagcaacaccttcttcacgaggcagacctcactagttccactgagcgtcagaccccgtagaaaagatcaaaggatcttcttgagatcctttttttctgcgcgtaatctgctgcttgcaaacaaaaaaaccaccgctaccagcggtggtttgtttgccggatcaagagctaccaactctttttccgaaggtaactggcttcagcagagcgcagataccaaatactgtccttctagtgtagccgtagttaggccaccacttcaagaactctgtagcaccgcctacatacctcgctctgctaatcctgttaccagtggctgctgccagtggcgataagtcgtgtcttaccgggttggactcaagacgatagttaccggataaggcgcagcggtcgggctgaacggggggttcgtgcacacagcccagcttggagcgaacgacctacaccgaactgagatacctacagcgtgagcattgagaaagcgccacgcttcccgaagggagaaaggcggacaggtatccggtaagcggcagggtcggaacaggagagcgcacgagggagcttccagggggaaacgcctggtatctttatagtcctgtcgggtttcgccacctctgacttgagcgtcgatttttgtgatgctcgtcaggggggcggagcctatggaaaaacgccagcaacgcggcctttttacggttcctggccttttgctggccttttgctcacatgttctttcctgcgttatcccctgattctgtggataaccgtattaccgcctttgagtgagctgataccgctcgccgcagccgaacgaccgagcgcaacgcgtgcggccgcggtacccggggatcctctagagtcgaccaccaagggcaccatctctgcttgggccaccccgttggccgcagccagctcgctgagagccgtgaacgacagggcgaacgccagcccgccgacggcgagggttccgaccgctgcaactcccggtgcaaccttgtcccggtctattctcttcactgcaccagctccaatctggtgtgaatgcccctcgtctgttcgcgcaggcggggggctctattcgtttgtcagcatcgaaagtagccagatcagggatgcgttgcaaccgcgtatgcccaggtcagaagagtcgcacaagagttgcagacccctggaaagaaaaatggccagagggcgaaaacaccctctgaccagcggagcgggcgacgggaatcgaacccgcgtagctagtttggaagaatgggtgtctgccgaccacatatgggccggtcaagataggtttttaccccctctcggctgcatcctctaagtggaaagaaattgcaggtcgtagaagcgcgttgaagcctgagagttgcacaggagttgcaacccggtagccttgttcacgacgagaggagacctagttggcacgtcgcggatggggatcgctgaagactcagcgcagcgggaggatccaagcctcatacgtcaacccgcaggacggtgtgaggtactacgcgctgcagacctacgacaacaagatggacgccgaagcctggctcgcgggcgagaagcggctcatcgagatggagacctggacccctccacaggaccgggcgaagaaggcagccgccagcgccatcacgctggaggagtacacccggaagtggctcgtggagcgcgacctcgcagacggcaccagggatctgtacagcgggcacgcggagcgccgcatctacccggtgctaggtgaagtggcggtcacagagatgacgccagctctggtgcgtgcgtggtgggccgggatgggtaggaagcacccgactgcccgccggcatgcctacaacgtcctccgggcggtgatgaacacagcggtcgaggacaagctgatcgcagagaacccgtgccggatcgagcagaaggcagccgatgagcgcgacgtagaggcgctgacgcctgaggagctggacatcgtcgccgctgagatcttcgagcactaccggatcgcggcatacatcctggcgtggacgagcctccggttcggagagctgatcgagcttcgccgcaaggacatcgtggacgacggcatgacgatgaagctccgggtgcgccgtggcgcttcccgcgtggggaacaagatcgtcgttggcaacgccaagaccgtccggtcgaagcgtcctgtgacggttccgcctcacgtcgcggagatgatccgagcgcacatgaaggaccgtacgaagatgaacaagggccccgaggcattcctggtgaccacgacgcagggcaaccggctgtcgaagtccgcgttcaccaagtcgctgaagcgtggctacgccaagatcggtcggccggaactccgcatccacgacctccgcgctgtcggcgctacgttcgccgctcaggcaggtgcgacgaccaaggagctgatggcccgtctcggtcacacgactcctaggatggcgatgaagtaccagatggcgtctgaggcccgcgacgaggctatcgctgaggcgatgtccaagctggccaagacctcctgaaacgcaaaaagcccccctcccaaggacactgagtcctaaagaggggggtttcttgtcagtacgcgaagaaccacgcctggccgcgagcgccagcaccgccgctctgtgcggagacctgggcaccagccccgccgccgccaggagcattgccgttcccgccagaaatctagaggtgaccacaacgacgcgcccgctttgatcggggacgtctgcggccgaccatttacgggtcttgttgtcgttggcggtcatgggccgaacatactcacccggatcggagggccgaggacaaggtcgaacgaggggcatgacccggtgcggggcttcttgcactcggcataggcgagtgctaagaataacgttggcactcgcgaccggtgagtgctaggtcgggacggtgaggccaggcccgtcgtcgcagcgagtggcagcgaggacaacttgagccgtccgtcgcgggcactgcgcccggccagcgtaagtagcggggttgccgtcacccggtgacccccggtttcatccccgatccggaggaatcacttcgcaatggccaagacaattgcggatccagctgcagaattcgacgaatttgaaatgataaagagaaacacatctgaaattatcagcgaggaagagttaagagaggttttaaaaaaagatgaaaaatctgctctgataggttttgaaccaagtggtaaaatacatttagggcattatctccaaataaaaaagatgattgatttacaaaatgctggatttgatataattatattgttggctgatttacacgcctatttaaaccagaaaggagagttggatgagattagaaaaataggagattataacaaaaaagtttttgaagcaatggggttaaaggcaaaatatgtttatggaagttcgttccagcttgataaggattatacactgaatgtctatagattggctttaaaaactaccttaaaaagagcaagaaggagtatggaacttatagcaagagaggatgaaaatccaaaggttgctgaagttatctatccaataatgcaggttaatcctcttcattatgagggcgttgatgttgcagttggagggatggagcagagaaaaatacacatgttagcaagggagcttttaccaaaaaaggttgtttgtattcacaaccctgtcttaacgggtttggatggagaaggaaagatgagttcttcaaaagggaattttatagctgttgatgactctccagaagagattagggctaagataaagaaagcatactgcccagctggagttgttgaaggaaatccaataatggagatagctaaatacttccttgaatatcctttaaccataaaaaggccagaaaaatttggtggagatttgacagttaatagctatgaggagttagagagtttatttaaaataagggattgcatccaatggatttaaanatgctgtagctgaagaacttataaagattttagagccaattagaaagagattataaaagcttatcgatgtcgacgtagttaactagcgtacgatcgactgccaggcatcaaataaaacgaaaggctcagtcgaaagactgggcctttcgttttatctgttgtttgtccggccatcatggccgcggtgatca
