# Supplementary figures and images for: Genetic Incorporation of Unnatural Amino Acids into Proteins in Mycobacterium tuberculosis
Source: PLoS One. 2010 Feb 22;5(2):e9354. doi: 10.1371/journal.pone.0009354 (PMC2825273; doi:10.1371/journal.pone.0009354)

A

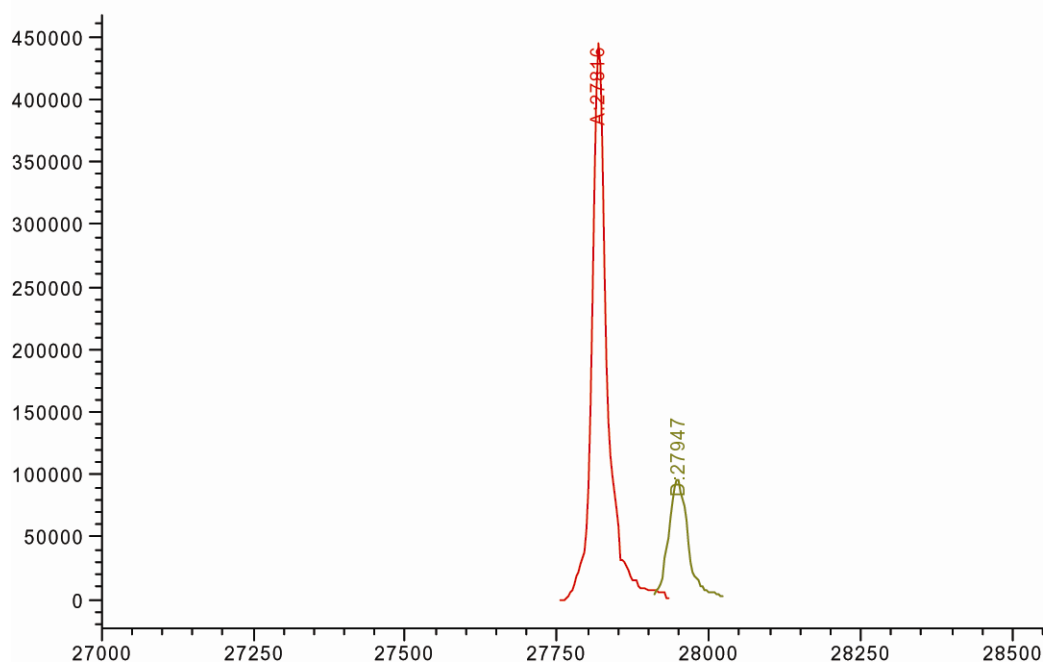

B

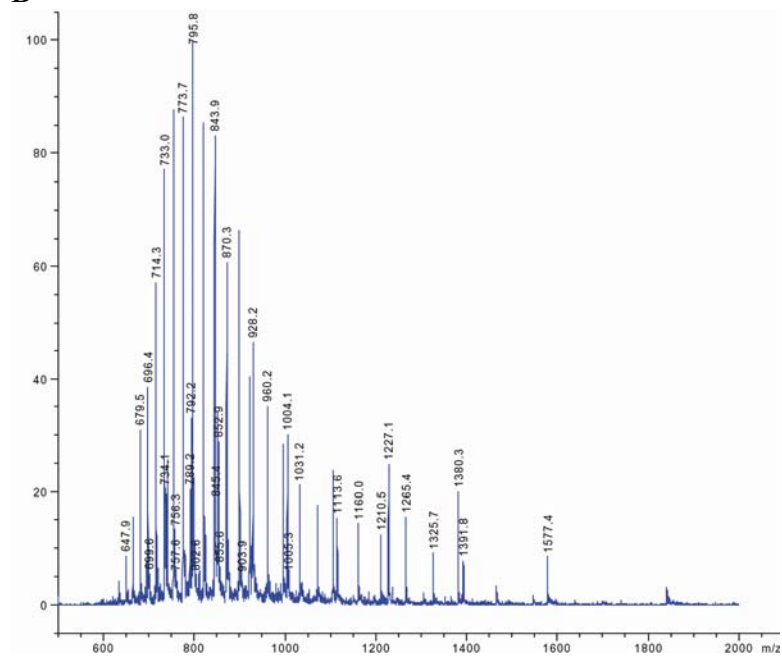

Supplement: Figure S1 — A. Deconvoluted ESI-MS spectra of the GFP mutant Tyr151→pIpa. Expected mass is 27815 Da; observed masses are 27816 Da and 27947 Da (with N-terminal methionine). B. Full ESI-MS spectra of the GFP mutant Tyr151→pIpa. (0.05 MB PDF) [file pone.0009354.s002.pdf]

A

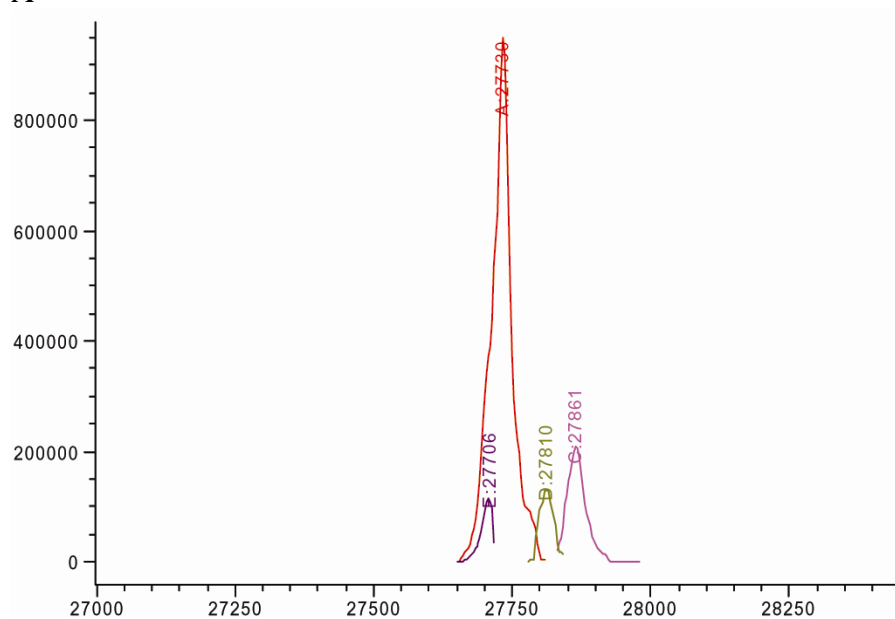

B

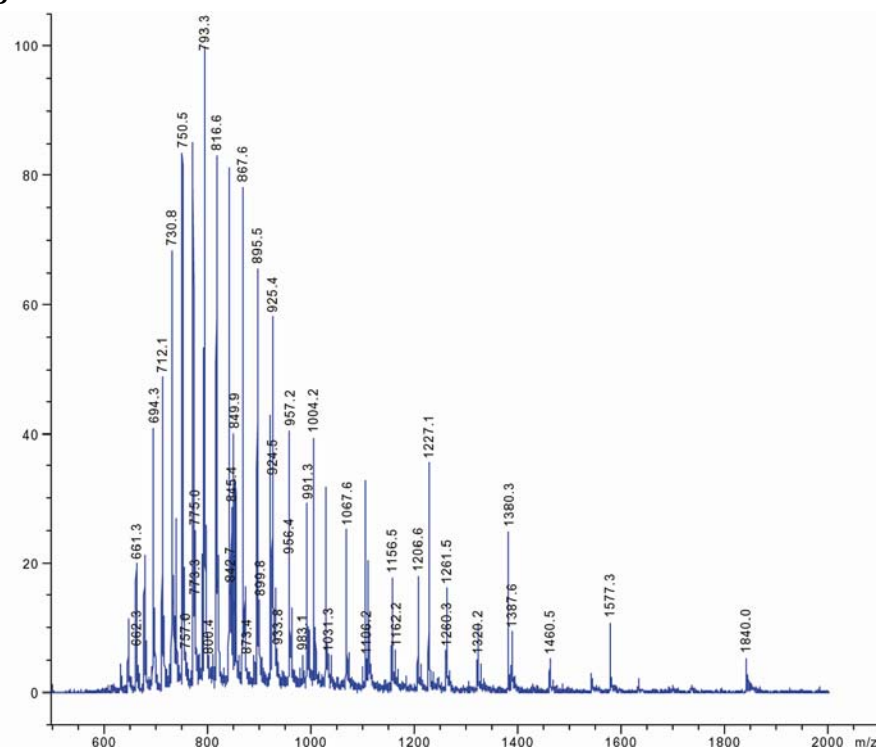

Supplement: Figure S2 — Deconvoluted ESI-MS spectra of the GFP mutant Tyr151→pAzpa. Expected mass is 27733 Da; observed masses are 27730 Da and 27861 Da (with N-terminal methionine); the observed mass 27706 corresponds to the mass of GFP mutant Tyr151→pAzpa after the azido group is reduced to amine by photo activation. The mass peak 27810 is not corresponding to the natural amino acid incorporated GFP and is most likely impurity. B. Full ESI-MS spectra of the GFP mutant Tyr151→pAzpa. (0.06 MB PDF) [file pone.0009354.s003.pdf]

A

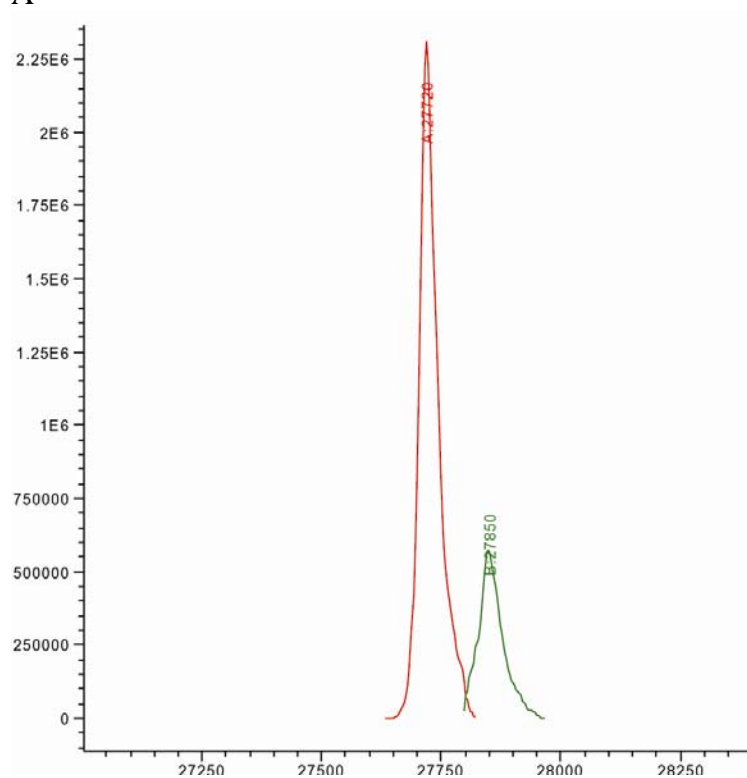

B

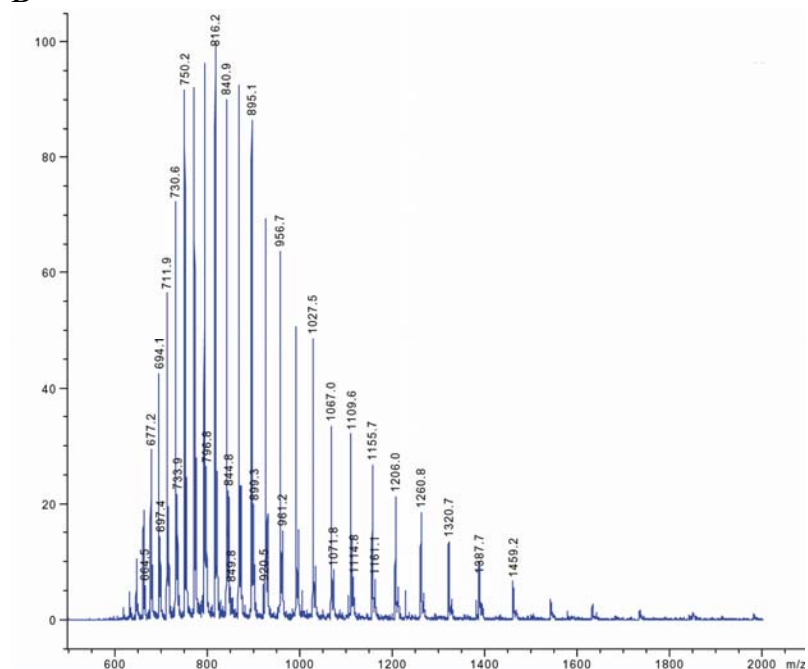

Supplement: Figure S3 — Deconvoluted ESI-MS spectra of the GFP mutant Tyr151→pBO2pa. Expected mass is 27718 Da; observed masses are 27720 Da and 27850 Da (with N-terminal methionine). B. Full ESI-MS spectra of the GFP mutant Tyr151→pBO2pa. (0.06 MB PDF) [file pone.0009354.s004.pdf]

A

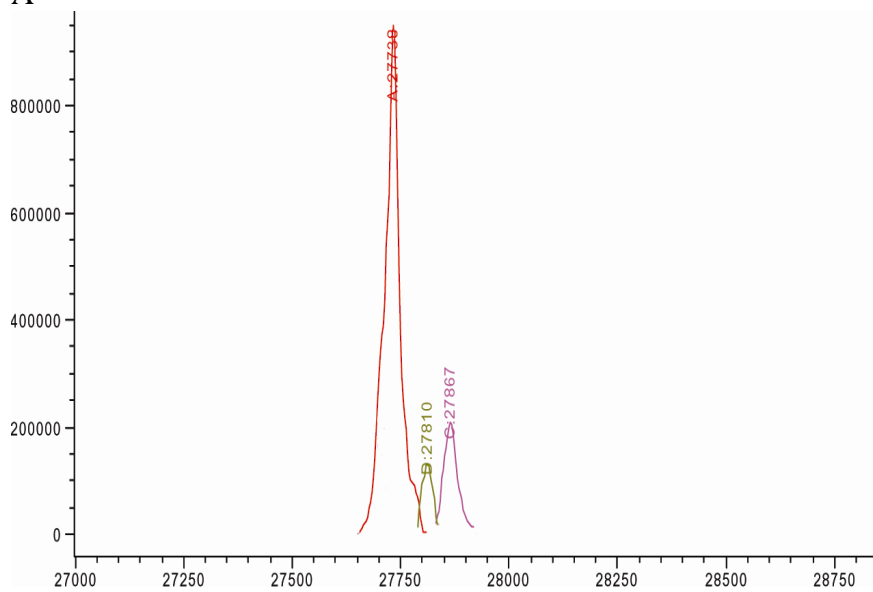

B

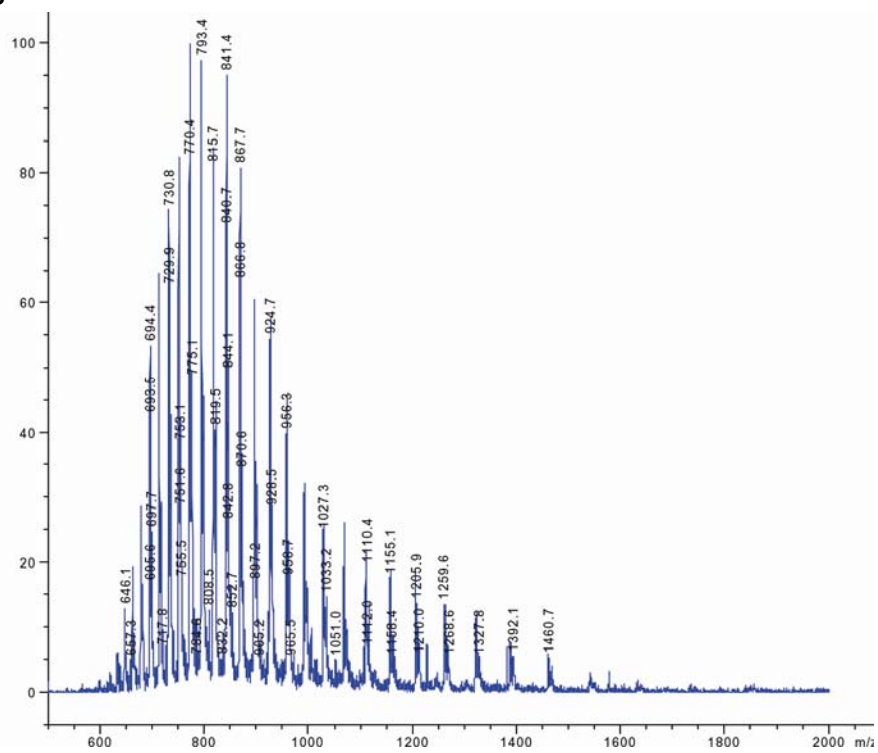

Supplement: Figure S4 — Deconvoluted ESI-MS spectra of the GFP mutant Tyr151→pNO2pa. Expected mass is 27737 Da; observed masses are 27738 Da and 27867 Da (with N-terminal methionine). The mass peak 27810 is not corresponding to the natural amino acid incorporated GFP and is most likely impurity. B. Full ESI-MS spectra of the GFP mutant Tyr151→pNO2pa. (0.06 MB PDF) [file pone.0009354.s005.pdf]

A

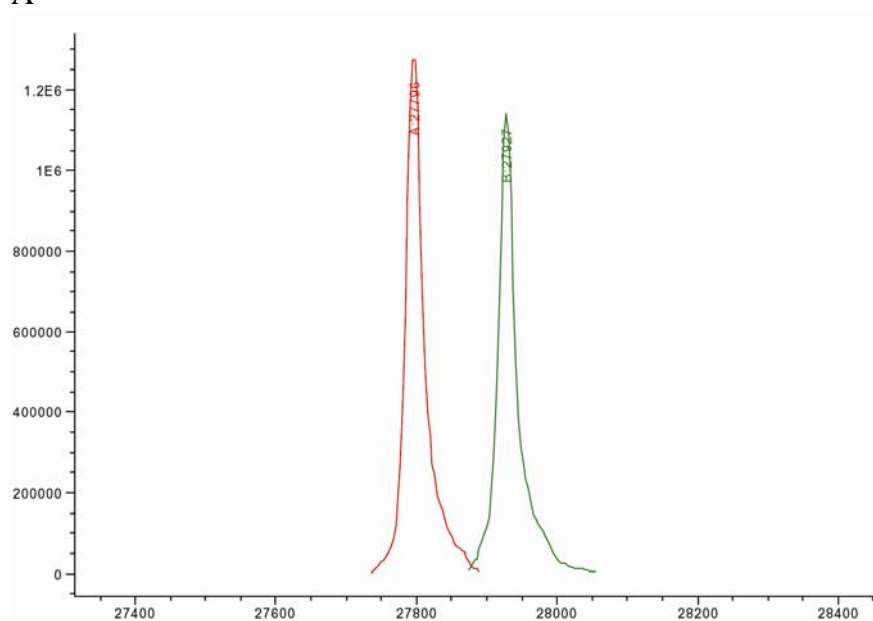

B

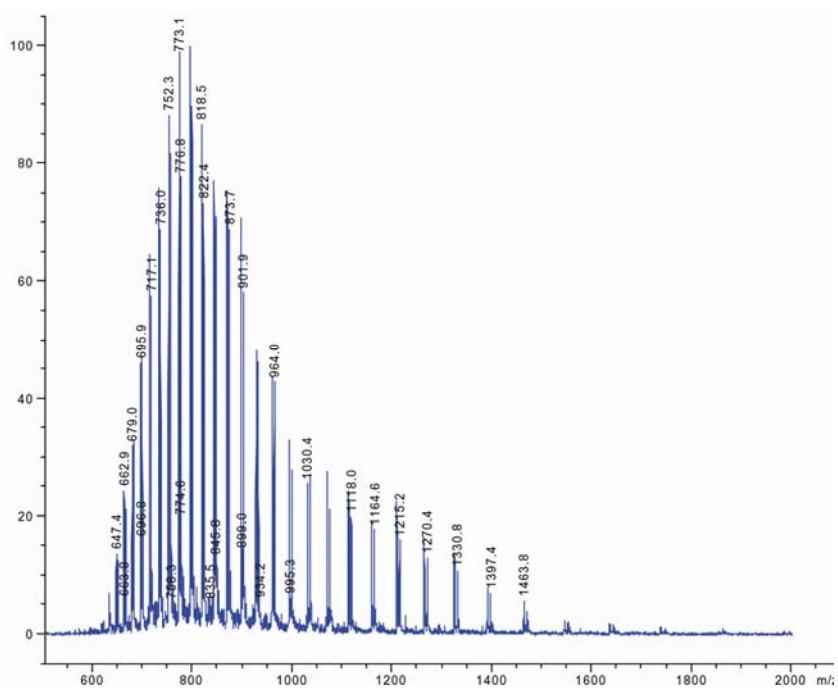

Supplement: Figure S5 — Deconvoluted ESI-MS spectra of the GFP mutant Tyr151→pBpa. Expected mass is 27796 Da; observed masses are 27795 Da and 27927 Da (with N-terminal methionine). B. Full ESI-MS spectra of the GFP mutant Tyr151→pBpa. (0.06 MB PDF) [file pone.0009354.s006.pdf]

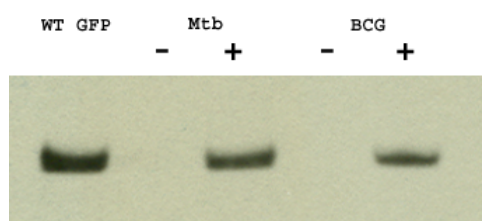

Supplement: Figure S6 — Western blot analysis of GFP expression in Mtb and BCG cells that were cotransformed with pSMT-MjtRNA-GFP115TAG and pMV361-MjpIpaRS. The first lane is wild type GFP expressed in M. smegmatis. The following lanes are expression of GFP151TAG suppressed by MjpIpaRS in the presence (+) and absence (−) of 1 mM pIpa. A 60 µg aliquot of cell lysate for each reaction (10 µg for the WT) was analyzed with anti-His-HRP. (0.08 MB PDF) [file pone.0009354.s007.pdf]

A.

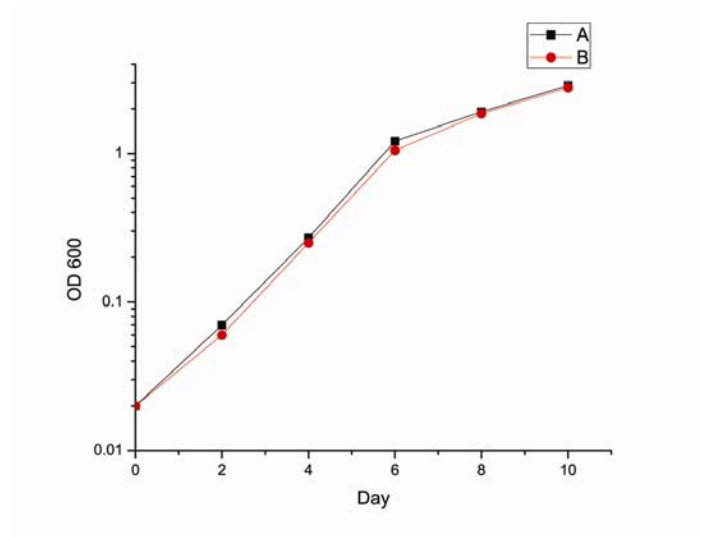

B.

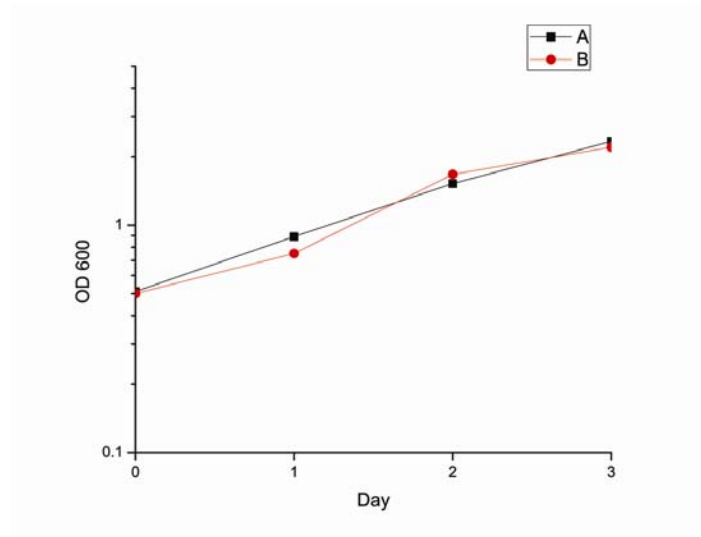

Supplement: Figure S7 — Growth of pIpa-GFP151TAG-H37Ra. A. Growth rates of wild type H37Ra (black dots) and strain pIpa-GFP151TAG-H37Ra (red dots) in 7H9 media. B. Growth rates of wild type H37Ra (black dots) and strain pIpa-GFP151TAG-H37Ra (red dots) in 7H9 media treated with 1 mM pIpa. (0.03 MB PDF) [file pone.0009354.s008.pdf]
